# Silica nanoparticles induce neurodegeneration-like changes in behavior, neuropathology, and affect synapse through MAPK activation

**DOI:** 10.1186/s12989-018-0263-3

**Published:** 2018-07-03

**Authors:** Ran You, Yuen-Shan Ho, Clara Hiu-Ling Hung, Yan Liu, Chun-Xia Huang, Hei-Nga Chan, See-Lok Ho, Sheung-Yeung Lui, Hung-Wing Li, Raymond Chuen-Chung Chang

**Affiliations:** 1 Laboratory of Neurodegenerative Diseases, School of Biomedical Sciences, LKS Faculty of Medicine, The University of Hong Kong, Pokfulam, Hong Kong, SAR China; 2 School of Nursing, Faculty of Health and Social Sciences, The Hong Kong Polytechnic University, Hung Hom, Kowloon, Hong Kong, SAR China; 3 State Key Laboratory of Brain and Cognitive Sciences, The University of Hong Kong, Pokfulam, Hong Kong, SAR China; 4 Department of Chemistry, Hong Kong Baptist University, Kowloon Tong, Hong Kong, SAR China; 5 School of Biomedical Sciences, Rm. L4-49, Laboratory Block, Faculty of Medicine Building, 21 Sassoon Road, Pokfulam, Hong Kong; 6 Present address: Nanjing Key Laboratory of Pediatrics, Children’s Hospital of Nanjing Medical University, Nanjing, 210008 China

**Keywords:** Silica nanoparticles, Neurodegeneration, Behavior, Synapse

## Abstract

**Background:**

Silica nanoparticles (SiO_2_-NPs) are naturally enriched and broadly utilized in the manufacturing industry. While previous studies have demonstrated toxicity in neuronal cell lines after SiO_2_-NPs exposure, the role of SiO_2_-NPs in neurodegeneration is largely unknown. Here, we evaluated the effects of SiO_2_-NPs-exposure on behavior, neuropathology, and synapse in young adult mice and primary cortical neuron cultures.

**Results:**

Male C57BL/6 N mice (3 months old) were exposed to either vehicle (sterile PBS) or fluorescein isothiocyanate (FITC)-tagged SiO_2_-NPs (NP) using intranasal instillation. Behavioral tests were performed after 1 and 2 months of exposure. We observed decreased social activity at both time points as well as anxiety and cognitive impairment after 2 months in the NP-exposed mice. NP deposition was primarily detected in the medial prefrontal cortex and the hippocampus. Neurodegeneration-like pathological changes, including reduced Nissl staining, increased tau phosphorylation, and neuroinflammation, were also present in the brains of NP-exposed mice. Furthermore, we observed NP-induced impairment in exocytosis along with decreased synapsin I and increased synaptophysin expression in the synaptosome fractions isolated from the frontal cortex as well as primary neuronal cultures. Extracellular signal-regulated kinase (ERK) and c-Jun N-terminal kinase (JNK) were also activated in the frontal cortex of NP-exposed mice. Moreover, inhibition of ERK activation prevented NP-mediated changes in exocytosis in cultured neurons, highlighting a key role in the changes induced by NP exposure.

**Conclusions:**

Intranasal instillation of SiO_2_-NPs results in mood dysfunction and cognitive impairment in young adult mice and causes neurodegeneration-like pathology and synaptic changes via ERK activation.

**Electronic supplementary material:**

The online version of this article (10.1186/s12989-018-0263-3) contains supplementary material, which is available to authorized users.

## Background

Neurodegenerative diseases, including Alzheimer’s disease (AD), Parkinson’s disease (PD), amyotrophic lateral sclerosis, and frontotemporal dementia (FTD), affect a large and ever-growing population globally [1]. AD, for example, is the major cause of dementia in the elderly and is estimated to affect approximately 24 million people worldwide [2]. As this number is expected to double every 20 years until 2040, continued research to understand the causes as well as to elucidate drug targets is essential to enhance patient care and decrease the economic burden on both individuals and society.

Neurodegenerative diseases feature an assortment of clinical symptoms, including devastating changes in behavior and cognition. Collectively, neurodegenerative diseases are known to have convergent pathological markers, featured with selective dysfunction and loss of synapses and neurons, protein aggregates, and neuroinflammation [3–5]. These changes eventually disrupt connectivity in the neuronal circuitry of the brain [6, 7]. In AD and other neurodegenerative diseases, these changes most notably affect the hippocampus and frontal cortex, which are the two major brain regions responsible for cognitive function [6]. The hippocampus mainly regulates learning and memory, while the frontal cortex regulates working memory, higher levels of intelligence, and emotions such as social interaction [6] and anxiety [8]. Therefore, the impaired structural and functional integrity of the neuronal and synaptic networks in the hippocampus and/or the frontal cortex are typically manifested as behavioral symptoms, such as progressive cognitive decline and mood dysfunction, in patients with neurodegenerative diseases [9]. While both pathological and behavioral symptoms have been widely documented, pathogenesis of many neurodegenerative disease is largely unknown and often sporadic, a characteristic that is closely related to long-term exposure to environmental risk factors [10, 11]. Therefore, it is of vital importance to identify the environmental causes of neurodegeneration.

Silica nanoparticles (SiO_2_-NPs) are one of the most broadly exploited nanomaterials and have been utilized in a variety of industries [12–15]. They are also the most common component in a number of airborne pollutants, including mineral dust and particulate matter (PM), found in ambient air as well as circulated air in households and workplaces. Excessive exposure to SiO_2_-NPs has been shown to cause numerous health issues in susceptible cohorts, such as workers in industrial fields [16, 17]. In fact, recent studies have indicated that the brain may be a potential target for SiO_2_-NPs. After inhalation, SiO_2_-NPs penetrate the epithelium of the respiratory tract and are then translocated to the brain via either the circulatory system or the olfactory nerve [18]. Once in the brain, these nanoparticles may be toxic to neurons and even affect behavior. For example, in an in vitro study using multiple neuroblastoma cell lines, SiO_2_-NPs dose-dependently induced cytotoxicity and AD-like pathology [19]. SiO_2_-NPs were also shown to induce PD-like behavioral changes in zebrafish [20]. While these findings suggest that respiratory tract exposure to SiO_2_-NPs could be a risk factor for neurodegeneration, this has not been fully explored, and the mechanisms underlying these effects are largely unknown.

In the present study, we evaluated the role of amorphous SiO_2_-NPs through respiratory tract in the development of neurodegeneration-like changes in behavior and pathology in young adult mice. We used fluorescein isothiocyanate (FITC)-tagged SiO_2_-NPs (FITC-SiO_2_-NPs, NP), which have similar toxicity as untagged SiO_2_-NPs [21, 22], and investigated their longitudinal effects on mood and cognition. To further investigate the mechanisms underlying the behavioral changes in the NP-exposed mice, we first examined the deposition of NP in the medial prefrontal cortex (mPFC) as well as the cornu ammonis area (CA)1, CA3, and dentate gyrus (DG) in the hippocampus, and then documented the histological changes in the neurons and microglia in these regions. The associated pathological changes, including hyperphosphorylated tau, synaptic endocytosis and exocytosis, and synaptic density, were also examined. The observed changes in the synapses were then confirmed with 14-day primary cultures of cortical neurons exposed to NP for 48 h. We also studied the implication of two mitogen-activated protein kinases (MAPKs), i.e. extracellular signal-regulated kinase (ERK) and c-Jun N-terminal kinase (JNK), in the observed detrimental effects of NP on synapse. To our knowledge, this is the first detailed evaluation of the adverse effects of SiO_2_-NPs on behavior and in the brain, particularly with regards to the role of MAPKs activation in the NP-exposed synapse.

## Results

### Characterization of the FITC-SiO_2_-NPs

**Fig. 1:**
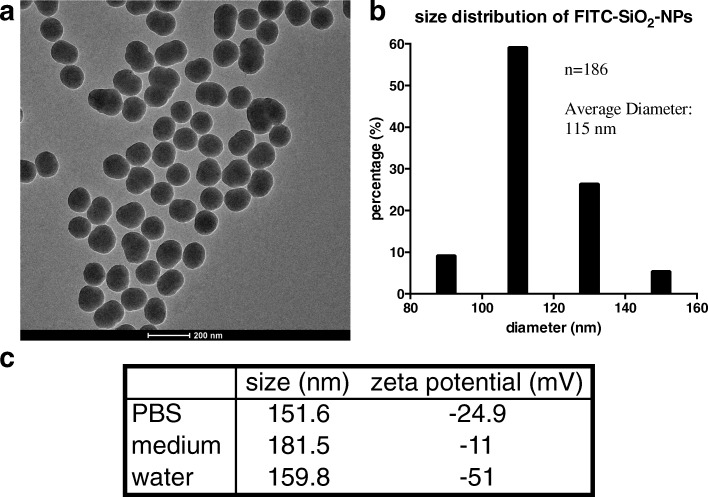
Characterization of FITC-SiO_2_-NPs via TEM (**a**, **b**) and DLS (**c**). **a** Representative TEM image of the constructed NPs. Scale bar: 200 nm. **b** Size distribution of the NPs. A total of 186 particles were measured in the TEM images. **c** Hydrodynamic size and zeta potential of the NP in PBS, culture medium, and water as determined by DLS

The morphology and size of the NP were characterized by transmission electron microscopy (TEM) (Fig. 1a). According to our data, these NP have an average diameter of 115 nm (Fig. 1b). Since the NP used in this study were suspended in aqueous fluids, namely phosphate buffered saline (PBS) and cortical neuron cell culture medium, we evaluated their hydrodynamic properties in these two fluids as well as water using dynamic light scattering (DLS) (Fig. 1c). The mean hydrodynamic diameter of the NP in water was 159.8 nm, while the zeta potential was − 51 mV. Notably, the NP had a similar hydrodynamic diameter and zeta potential in both PBS and in culture medium compared to that observed in water.

### FITC-SiO_2_-NPs decreased social interaction activity, induced anxiety and cognitive impairment

**Fig. 2:**
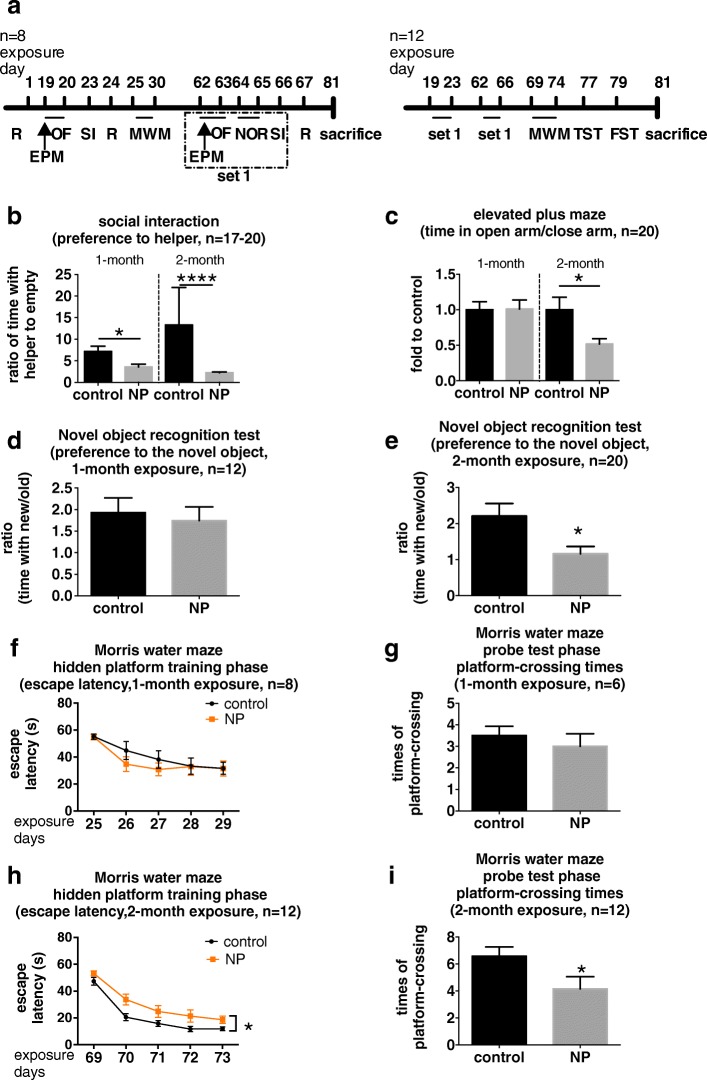
Effects of NP on mood and cognition in mice. **a** Experimental design for NP administration and behavioral tests. Male C57BL/6 N mice (3 months old) were administered PBS or NP (final dosage 8 mg/kg) via intranasal instillation once per day for 81 days. Behaviors were tested according to the plan. Abbreviations: R, rotarod test; OF, open field test; EPM, elevated plus maze test; NOR, novel object recognition test; SI, social interaction test; MWM, Morris water maze test; TST, tail suspension test; FST, forced swimming test. **b** SI activity (ratio of juvenile helper to empty chamber interaction,T_helper_/T_empty_) was tested at days 23 (1 month time point, left) and 66 (2 month time point, right) and analyzed with an unpaired Student’s *t*-test and unpaired non-parametric Mann-Whitney test, respectively. **c** EPM was used to evaluate anxiety, presented as the ratio of time spent in open arms to closed arms (T_open_/T_close_), at days 19 (1 month time point, left) and 62 (2 month time point, right) and analyzed with unpaired Student’s *t*-tests. The effects of NP exposure on cognition were tested with the NOR (**d**, 1 month; **e**, 2 months) and the MWM (**f** and **g**, training and probe phase at 1 month; **h** and **i**, training and probe phase at 2 months). For the NOR, memory is demonstrated by the ratio of time interacting with new object to that interacting with the old object (T_new_/T_old_). Data from 1 month and 2 months were analyzed by unpaired Student’s *t*-tests, and unpaired Mann-Whitney test, respectively. For the MWM, the learning curve was plotted using the average escape latency per day in the training phase and was analyzed using a repeated measures two-way ANOVA. Spatial memory, represented by the platform-crossing time during the probe phase, was analyzed using unpaired Mann-Whitney tests. * *p* < 0.05, **** *p* < 0.0001, compared to controls. *n* = 8, 12, or 20, indicates that each group has the same number of mice, while *n* = 17–20 means that all groups included 20 mice except the 1 month control group which only had 17 as 3 videos were accidentally lost

Motor function, mood, and cognition of mice exposed to NP or PBS (control) were tested in a battery of behavioral tests (outlined in Fig. 2a) after 1 and 2 months.

#### Locomotor and motor function

By measuring the distance the mice traveled in an open field test, we found that the locomotor function of the NP-exposed mice was not significantly different from that of the control mice at either time point (Additional file 1: Figure S1a). Furthermore, we also observed that the motor function of the NP-exposed mice, determined by measuring the time spent on an accelerating rotarod, was not significantly different from that of the control mice (Additional file 1: Figure S1b). These results indicated that the NP-exposed mice were as physically capable as the control mice. Thus, all of the mice, regardless of treatment, could accomplish the behavioral tests without any physical bias.

#### Mood

After both 1 month and 2 months of NP exposure, the mice showed a significant decrease in social interaction activity compared to the controls, as highlighted by results of the three-chambered social interaction test (1 month: − 3.57 fold, *p* = 0.0120; 2 months: − 11.06 fold, *p* < 0.0001; Fig. 2b). These data indicated that NP could be causing mood dysfunction in the exposed mice. Notably, anxiety and depression both involve social activity dysfunction and are common mood disorders observed in patients with neurodegeneration [23]. Therefore, we also investigated the effects of NP exposure on anxiety and depression using the elevated plus maze test and two despair tests, i.e. the forced swimming test and tail suspension test, respectively. In the elevated plus maze test, we found that the mice showed anxious behavior after 2 months of NP exposure (2 months: − 48.26%, *p* = 0.0163; Fig. 2c), but not at the earlier time point (Fig. 2c). Interestingly, similar trend was found in the open field test, which indicated anxiety by measuring the time spent in the central area. However, the difference between the two groups at 2 months time point was not statistically significant (Additional file 1: Figure S1c). During the despair tests, no significant differences were observed in immobility time during the forced swimming test (Additional file 1: Figure S1d) or the tail suspension test (Additional file 1: Figure S1e), suggesting that NP did not induce depression in mice.

#### Cognition

In the novel object recognition test, we found that the mice did not display any cognitive impairment after 1 month of NP exposure (Fig. 2d), but showed significantly impaired short-term memory after 2 months of exposure (− 1.044 fold, *p* = 0.0154; Fig. 2e), which was highlighted by their failure to recognize a novel object. Consistent with these findings, in the Morris water maze test, the mice exposed to NP for 1 month did not display any significant changes in learning or memory (Fig. 2f & g). However, after 2 months of NP exposure, the mice showed impaired spatial learning and memory in the Morris water maze test, which was demonstrated by their longer escape latency in the hidden-platform training phase (repeated measures two-way ANOVA, time factor: F(4,44) = 106.8, *p* < 0.0001; treatment factor: F(1,11) = 5.890, *p* = 0.0336; interaction: F(4,44) = 0.7856, *p* = 0.5407; Fig. 2h) as well as the fewer number of returns to the original platform position during the probe test (− 2.417 times, *p* = 0.0476, Fig. 2i). Notably, we did not observe a significant difference in quadrate preference during the probe test (Additional file 1: Figure S1f & g), indicating that both the control and NP-exposed groups searched for the platform using spatial memory rather than associative memory.

### Localization of FITC-SiO_2_-NPs in the brain

**Fig. 3:**
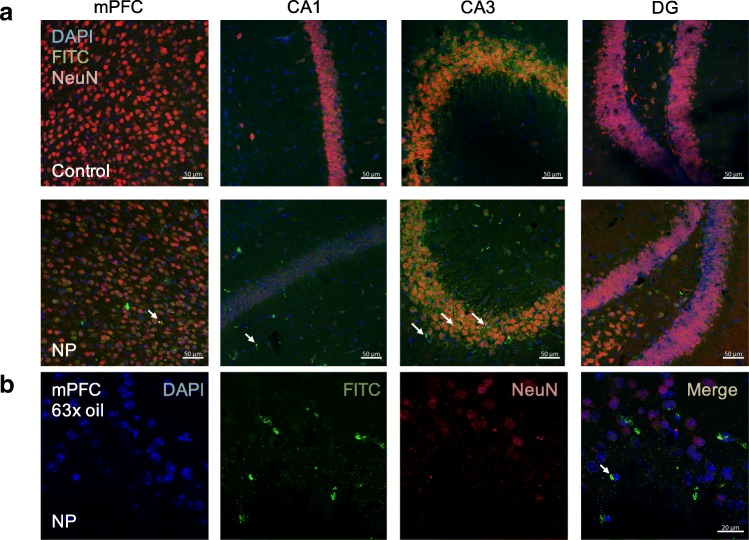
Deposition of NP in the brain. After the mice were intranasally instilled with either vehicle (sterile PBS, control group) or NPs in PBS (8 mg/kg, NP group) once per day for 81 days, the mice were sacrificed and the brains were harvested, fixed, sectioned, and stained with anti-FITC antibody. Three non-overlapping fields in the mPFC as well as the CA1, CA3, and DG in the hippocampus were then imaged using confocal microscopy. **a** Representative images of the mPFC, CA1, CA3, and DG in the control group (upper panel) and NP group (middle panel) taken with a 20X objective lens. Scale bar: 50 μm. **b** Representative images of the mPFC in the NP group taken with a 63X oil lens. Scale bar: 20 μm. NP are highlighted by white arrows

Using immunofluorescent staining, we observed NP deposition in the mPFC (Fig. 3a and b) as well as the CA1 and CA3 in the hippocampus, but not in the DG (Fig. 3a).

### FITC-SiO_2_-NPs induced neurodegeneration-like pathological changes in the mPFC and the hippocampus

**Fig. 4:**
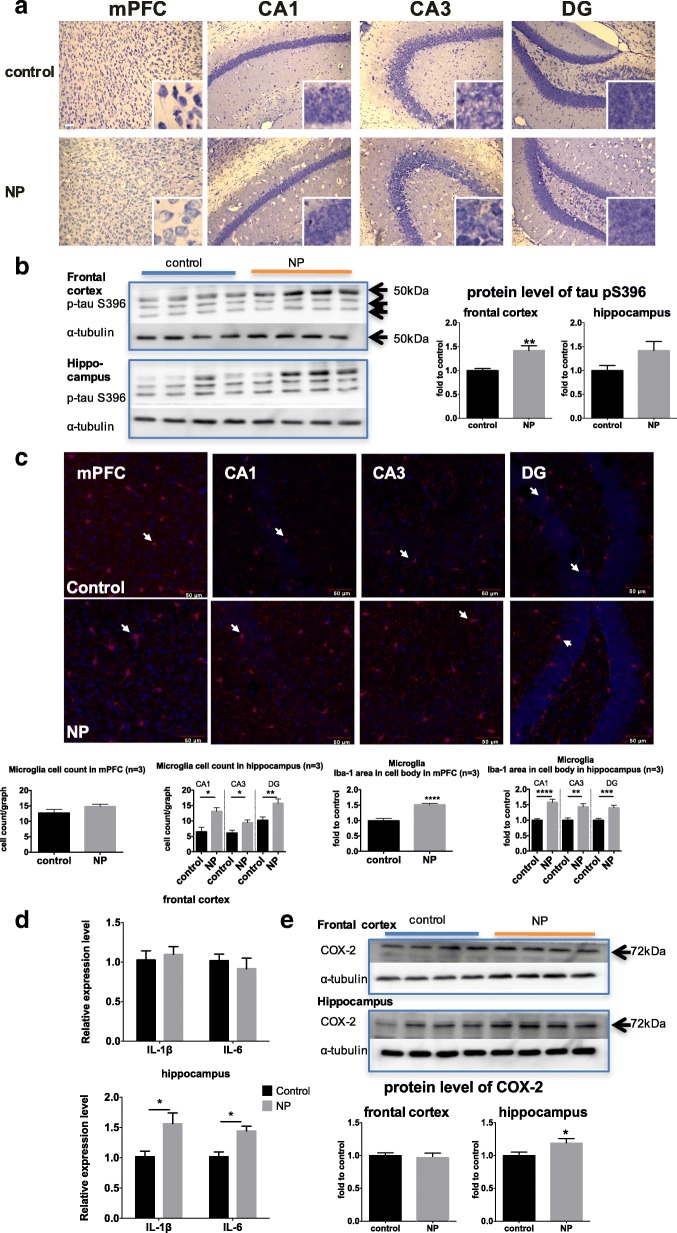
Neuropathological effects of NP in the brain. After the mice were intranasally instilled with either vehicle (sterile PBS, control group) or NP in PBS (8 mg/kg, NP group) once per day for 81 days, the mice were sacrificed and the brains were harvested. Half of the brain was fixed, sectioning, and used in Niss1 staining or Iba-1 immunofluorescent staining, while the frontal cortex and the hippocampus of the other half were homogenized. **a** Representative images of Nissl staining in the mPFC and the hippocampus. Zoom-in image demonstrating typical morphology in Nissl staining were presented in the bottom right corner of each image (*n* = 4 per group for mPFC, *n* = 3 per group for hippocampus). Scale bar: 50 μm. **b** Western blot analysis of tau phosphorylation at Serine 396 in the frontal cortex and hippocampus. Image shows four representative protein bands. Band intensity was normalized to that of α-tubulin and analyzed using unpaired Student’s *t*-tests (*n* = 8 for each group, except *n* = 7 for hippocampus control). **c** Representative images of Iba-1 (red) co-stained with DAPI (blue) in the mPFC and the hippocampus. Typical microglial morphology is highlighted with an arrow. The number and area of the cell bodies with high expression of Iba-1 in each sub-region (area of interest) were measured using Image J and analyzed using an unpaired Mann-Whitney test and an unpaired Student’s *t*-test, respectively (*n* = 3 per group). Scale bar: 50 μm. **d** mRNA expression of cytokines in the frontal cortex and the hippocampus by real time-PCR. Unpaired Student’s *t*-test was used as statistical analysis in each marker. (*n* = 6 per group) (**e**) Western blot analysis of COX-2 expression in the frontal cortex and hippocampus. Image shows four representative protein bands. Band intensity was normalized to that of α-tubulin and analyzed using unpaired Student’s *t*-tests (n = 8 for each group, except *n* = 7 for hippocampus control). For all analyses, * *p* < 0.05, ** *p* < 0.01, *** *p* < 0.001, **** *p* < 0.0001, compared to the controls

Nissl staining mainly reveals the soma of neurons, including the nucleoli and the rough endoplasmic reticulum which is also called Nissl bodies. In neurodegeneration, the morphology of neurons may change, featured with the diminish of Nissl bodies. Neurons in the mPFC of the control group showed normal morphology with the nucleus in the center and Nissl bodies in purple distributed in soma, whilst neurons in the NP group showed eccentric nucleus and profound lighter staining in the soma, indicating reduction and disperse of the Nissl bodies (Fig. 4a). However, no significant differences in the Nissl bodies were observed between the control and NP-exposed groups in any region of the hippocampus (Fig. 4a).

To further evaluate the pathology induced by NP treatment, we studied tau phosphorylation. Tau is a microtubule-associated protein that binds to and stabilizes microtubules. Moreover, increased tau phosphorylation is involved in the pathology of many neurodegenerative diseases, including AD and PD-related dementia [4]. Serine 396 is a common phosphorylation site in the tau protein that has been associated with neurodegeneration. In this study, tau phosphorylation at serine 396 was significantly increased in the frontal cortex of NP-exposed mice compared to the controls (41.84%, *p* = 0.0019; Fig. 4b). However, while an increase in tau phosphorylation at this site was also observed in the hippocampus of NP-exposed mice, this difference was not significant compared to the controls (Fig. 4b).

### FITC-SiO_2_-NPs induced neuroinflammation in the hippocampus

Neuroinflammation is a crucial pathological hallmark and mediator of neurodegenerative disease. The key players in this process are the activated microglia [24], which proliferate and express/secrete increased levels of pro-inflammatory cytokines, such as interleukin (IL)-1β and IL-6, as well as other inflammatory mediators, such as cyclooxygenase 2 (COX-2) [25]. After exposure to NP, the number of microglia, stained with Iba-1 antibody, was significantly increased in the hippocampus compared to the controls (CA1: 102.10%, *p* = 0.0186; CA3: 56.33%, p = 0.0186; DG: 54.22%, *p* = 0.0065; Fig. 4c). There was also an increase in cell body area in the mPFC (51.11%, *p* < 0.0001) and the hippocampus (CA1: 58.49%, p < 0.0001; CA3: 43.36%, *p* = 0.0017; DG: 40.23%, *p* = 0.0004) of NP group mice (Fig. 4c). These results indicated that NP induced microglial activation in these regions of the brain. Consistent with these data, we also observed a significant increase in the mRNA expression of IL-1β (60.69%, *p* = 0.0375; Fig. 4d) and IL-6 (41.71%, *p* = 0.0441; Fig. 4d), and in the protein level of COX-2 (19.21%, *p* = 0.0442; Fig. 4e) in the hippocampus of NP-exposed mice compared to controls. Microglial activation and the upregulation of pro-inflammatory markers in the hippocampus suggest that NP exposure induced neuroinflammation in this region of the brain.

### FITC-SiO_2_-NPs activated MAPKs

**Fig. 5:**
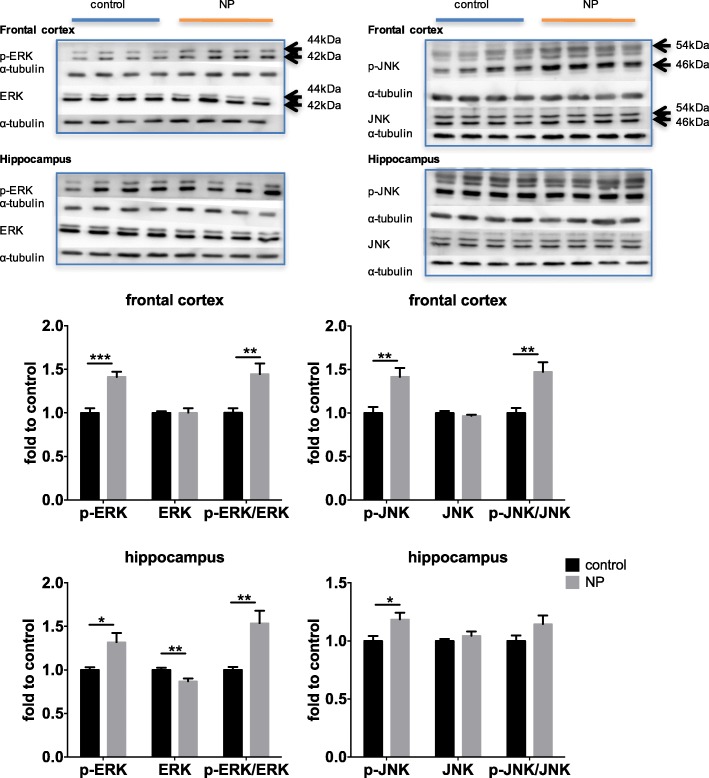
Effects of NP on MAPK activation in the brain. Western blot analysis of various MAPKs in total protein lysates isolated from the frontal cortex and hippocampus (*n* = 8 for each group, except *n* = 7 for hippocampus control). Images show four representative protein bands. Band intensity was first normalized to that of α-tubulin. Phosphorylation was shown by phosphorylation form normalized to total form. Data was analyzed using unpaired Student’s *t*-tests. *p < 0.05, **p < 0.01, *** *p* < 0.001 compared to the control

MAPKs respond to extracellular stimulation, and MAPK activation has been shown to be directly involved in tau phosphorylation and inflammation in the brain [26]. Thus, we evaluated the roles of three MAPKs, ERK, JNK, and p38, in the observed NP-induced changes in the brain. As shown in Fig. 5, ERK phosphorylation was significantly increased in both the frontal cortex (44.48%, *p* = 0.0051) and the hippocampus (53.66%, *p* = 0.0046) after NP exposure compared to the controls, while JNK phosphorylation was significantly increased primarily in the frontal cortex (41.50%, *p* = 0.0043). These results indicate that ERK and JNK signaling likely played a role in the NP-induced cellular changes in the brain. However, we did not observe any differences in p38 phosphorylation between the NP- and PBS-treated mice nor in the expression of cAMP responsive element binding protein (CREB), which is a downstream pro-survival signaling factor [27] (Additional file 1: Figure S2).

### FITC-SiO_2_-NPs impaired synaptic exocytosis in the frontal cortex and in primary neuronal cell cultures

**Fig. 6:**
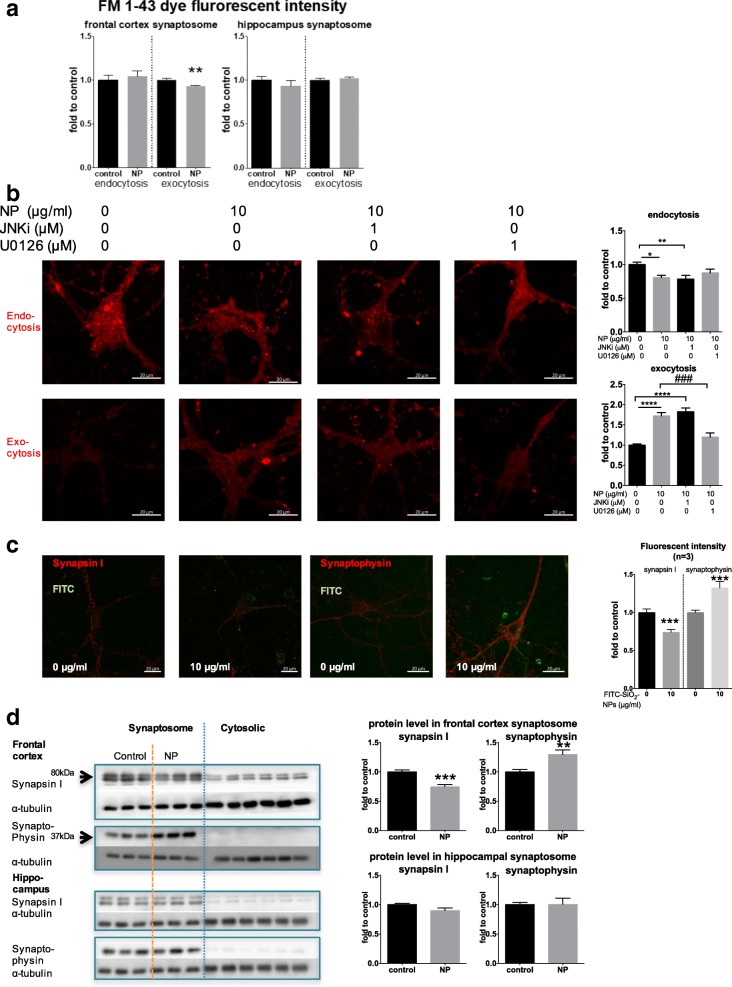
Effects of NP on synapse. After the mice were intranasally instilled with either vehicle (sterile PBS, control group) or NP in PBS (8 mg/kg, NP group) once per day for 81 days, the mice were sacrificed and the frontal cortex and hippocampus of the brains were harvested, followed by isolation of the synaptosome fraction. **a** An FM dye assay was performed to measure endocytosis and exocytosis in the synaptosome fraction from the frontal cortex and the hippocampus. Endocytosis was determined by the change in fluorescence intensity in the first step (F_endo_), while exocytosis was calculated using the change in fluorescence intensity in the second step with the equation (F_endo_ - F_exo_)/F_endo_. Data were normalized to the control group at each step and analyzed with unpaired Student’s *t*-tests (*n* = 8). **b** The effect of JNK and ERK inhibitors on endocytosis and exocytosis were evaluated using an FM dye assay. Primary neuronal cultures were treated with pharmacological inhibitors against JNK (JNKi) or ERK (U0126) for 1 h before NP exposure (48 h). Endocytosis and exocytosis levels were then determined/calculated as described in Methods section. Data were normalized to the control group at each step and analyzed by one-way ANOVA followed by Tukey’s Post hoc test (n = 4). Scale bar: 20 μm. **c** Representative images showing synapsin I and synaptophysin protein expression in 14-day primary cortical neuron cultures exposed to NP for 48 h (*n* = 3 independent batches of primary culture of cortical neurons). A total of 5 cells were imaged on each cover slip. Scale bar: 20 μm. **d** Western blot analysis of synapsin I and synaptophysin protein expression in the frontal cortex and hippocampus synaptosome fractions. Band intensity was normalized to that of α-tubulin and analyzed using unpaired Student’s *t*-tests (*n*=8). * *p* < 0.05, ** *p* < 0.01, *** *p* < 0.001, **** *p* < 0.0001, compared to the control; ### *p* < 0.001, compared to the NP group

Exocytosis and endocytosis are essential processes in synapse firing, allowing communication to occur between neurons. Using an FM dye assay to analyze the synaptosome fractions isolated from the frontal cortex, we found that exocytosis was significantly impaired in the NP-exposed mice compared to the controls (− 6.76%, *p* = 0.0092; Fig. 6a). However, neither endocytosis nor exocytosis was affected in the hippocampus (Fig. 6a).

We also evaluated endocytosis and exocytosis in primary cultures of cortical neurons. Notably, while the control group neurons efficiently loaded and released the FM 4–64 dye during endocytosis and exocytosis, respectively, these processes were impaired in the NP-exposed neurons (endocytosis: − 19.44%, *p* < 0.05; exocytosis: 72.33%, *p* < 0.0001; Fig. 6b). As endocytosis and exocytosis have also been linked to MAPKs function, we investigated the effects of blocking ERK and JNK activation on these synaptic vesicle transport processes. For this analysis, ERK inhibitor U0126 or JNK inhibitor II (JNKi, SP600125) was used to treat the primary cultures of cortical neurons 1 h prior to the 48 h NP exposure. While inhibition of JNK activation did not significantly alter the NP-mediated effects on endocytosis and exocytosis, inhibition of ERK activation significantly blocked the effects of NP exposure on exocytosis in the inhibitor-treated neurons compared to cells treated with NP alone (− 30.44%, *p* < 0.001 vs NP neurons; Fig. 6b). These results indicate that ERK activation may play a significant role in NP-induced synaptotoxicity.

### FITC-SiO_2_-NPs altered the expression of important synapse structural proteins

Synapsin I and synaptophysin are presynaptic proteins with different, but essential functions. We observed a decrease in synapsin I protein expression and an increase in synaptophysin protein expression in both the primary cortical neuron cultures as measured by immunofluorescence shown in Fig. 6c (synapsin I: − 26.04%, *p* = 0.0002; synaptophysin: 32.32%, *p* = 0.0009) and by Western-blot shown in Additional file 1: Figure S3 (synapsin I: − 14.18%, *p* = 0.0348; synaptophysin: 41.95%, *p* = 0.0096), and the synaptosome fraction of the frontal cortex in NP-exposed mice (synapsin I: − 25.18%, p = 0.0002; synaptophysin: 29.90%, *p* = 0.0038; Fig. 6d) compared to their respective controls. Thus, NP exposure appeared to alter synaptic structure.

## Discussion

Epidemiological and experimental studies have indicated that exposure to nano-scaled particles, varying from fine or ultrafine PM in airborne pollution to artificially engineered NP with different chemical compositions, may induce both cognitive impairment and/or mood dysfunction [28]. SiO_2_-NPs are a significant component of airborne PM, and exposure has been shown to cause a variety of health issues. Notably, amorphous silica is regarded as less toxic compared to crystalline silica, a well-known carcinogen [29]. However, the long-term effects of respiratory exposure to amorphous SiO_2_-NPs are not fully understood, particularly with regards to neurodegeneration. In the present study, we evaluated the role of amorphous SiO_2_-NPs in the development of neurodegeneration-like changes in brain pathology and behavior after intranasal instillation in young adult mice.

While this study is not the first to investigate the effects of nanoparticles on behavior, our investigation differs in three major aspects: age of the subjects, exposure time points, and the behavioral tests utilized. Notably, most of the existing studies investigating the effects of nanoparticles have been performed in young subjects, such as children [30, 31] or juvenile rodents (4 weeks old) [32–35], or in the elderly [36, 37], while the supporting evidence in adults is limited [38]. Thus, we focused on the impact of SiO_2_-NPs on young adult mice, whose well-developed brains should, in theory, be more resistant to stimulation [39]. However, our results demonstrate that NP could also affect this age group.

Furthermore, many of the previous studies have primarily focused on the short-term acute effects of SiO_2_-NPs. Indeed, intranasal instillation of SiO_2_-NPs once per day for 7 days was shown to induce a mild increase in the mRNA expression of pro-inflammatory cytokines in the hippocampus of rats [40]. Further studies investigating nanoparticles also indicated that the effect of short term exposure on behavior may be limited [41, 42]. Notably, one long-term study showed that exposing mice to PM2.5 for 10 months leads to profound behavioral changes in mood and cognition [32]. Therefore, in order to focus on the earliest, but significant, behavioral changes, we focused on the 1 and 2 month exposure time points in the present study.

To get a comprehensive understanding of the effects of SiO_2_-NPs exposure on behavior, we designed a battery of tests to evaluate multiple behavioral domains, including motor function, mood, and cognition. At least two tests were included for each, and although the tests for the same domain differ in sensitivity, motivation, and even behavior subcategory, the results revealed the same changes or at least the same trend in each domain. In fact, according to our results, social interaction activity was the most sensitive to NP treatment, being significantly reduced at both time points. At the 2 month time point, we also observed increased anxiety as well as impairments in memory and spatial learning. Therefore, it would appear that SiO_2_-NPs exposure causes significant changes in multiple behavioral domains, with most of these behaviors being affected after 2 months of exposure. Moreover, no significant differences were observed in the motor function tests, thus excluding any potential physical biases in the other tests.

One of the major concerns when studying behavior is the order of tests and the amount of rest between tests. In the present study, the tests were arranged under the principle of minimum interference and maximum habituation to decrease the adverse influence of one test on another. The mice were only exposed to one test per day, except for the elevated plus maze test, which followed the open field test as suggested in the literature [43]. Tests without externally driven forces, such as the open field test, elevated plus maze test, novel object recognition test, and social interaction test, were performed first. The interference could be further minimized by gradually increasing the level of stimulation involved in the tests, from none (open field test) to a novel object (novel object recognition test) and then to a novel mouse (social interaction test). As the motivation and stimulation levels in the rotarod test, Morris water maze test, and the despair tests gradually increase, these tests were performed later in the testing period and in this order. Interestingly, some test experiences may improve the stability and reliability of the outcomes of the following tests [43, 44]. This is largely because the initial experience could increase habituation. Sufficient habituation is of vital importance with regards to behavioral test performance, particularly for mice, which are highly sensitive to external stimulation. For example, the arena used for the open field test was also used for the later novel object recognition test and social interaction test, which could allow the animal to become familiar with their surroundings and focus on the tests.

In addition to behavior, we also evaluated the neuropathological changes induced by NP exposure. It was essential to first evaluate if the NP were translocated to the brain after intranasal instillation. To detect NP deposition in sections of the brain, which is a well-known autofluorescent tissue, we used an anti-FITC antibody to magnify the fluorescent intensity of the NP. This method allowed us to study the regional localization of the NP. Indeed, we detected the NP in sub-regions of the frontal cortex (mPFC) and the hippocampus (CA1 and CA3). These data are supported by a previous report showing similar deposition in the brains of rats exposed to ^125^I-labeled SiO_2_-NPs for 7 days [40]. Furthermore, this deposition pattern also correlated with the results of our behavioral tests as these two regions of the brain are critical for mood and cognition.

Surprisingly, we did not observe NP deposition in the DG, whose function is closely related to mood and cognition [45, 46]. However, neuroinflammation was observed in the DG as well as the CA1 and CA3 in the hippocampus, as indicated by the activation of microglia, and increased expression of IL-1β and IL-6 mRNA, and COX-2 protein in this region of the brain in NP-treated mice compared to the controls. These findings indicate that NP may affect the DG and other sub-regions of the brain by creating a pro-inflammatory environment.

Detecting pro-inflammatory changes in the brain after NP exposure was not altogether surprising as neuroinflammation was previously shown to be induced in both SiO_2_-NPs-exposed immortalized microglial cell cultures [47] and in the hippocampus after 7 days of SiO_2_-NPs exposure via intranasal instillation [40]. This type of NP-mediated inflammatory response is also strikingly similar to that observed in neurodegenerative diseases [3], particularly with regards to the downstream signaling molecules. Indeed, IL-1β is neurotoxic [48] and exacerbates neurodegenerative pathology and behaviors in multiple disease models, including AD [49], PD [50], poly I:C-induced neurodegeneration [51], and multiple sclerosis [52]. Further, the plasma levels of IL-6 have also been implicated as a biomarker for FTD [53] and AD [54]. COX-2 is known to be toxic to neurons and synapses [55] and has also been correlated with anxiety-like behavior [56] and cognitive impairment [57] in models of AD and PD [58]. Taken together, it is likely that neuroinflammation in the hippocampus contributes to the observed NP-induced behavioral changes.

Although neuroinflammation was mainly observed in the hippocampus, we also detected other neurodegeneration-like pathological changes including morphological changes in Nissl staining and increased phosphorylation of tau in the frontal cortex. Such a differentiated respond in these two regions of the brain may be due to their different neuroanatomy and intrinsic cellular responses to different stimuli. Similar differences were also found in response to other nanoparticles [59–61], toxins [62], and stress [63]. Noteworthy, NP exposure also induced synaptic changes in the frontal cortex. Synapses, especially the presynaptic terminals, are crucial for nanoparticles uptake into neurons because they are a location of active synaptic vesicle recycling via constant endocytosis and exocytosis [64]. Our results show that exocytosis was profoundly affected in both the synaptosome fraction isolated from the brains of NPs-exposed mice and in primary neuronal cultures exposed to NPs. Synaptic endocytosis and exocytosis are largely orchestrated by structural synaptic proteins and other subcellular structures, such as the cytoskeleton [64]. Therefore, functional changes in the synapses after NP treatment are often accompanied by structural changes. Indeed, we found that in addition to disrupted exocytosis, NP treatment also decreased synapsin I protein expression. Synapsin I is the primary linkage between the synaptic vesicles and the cytoskeleton. Decrease in synapsin I may lead to disrupted synaptic vesicle recycling [65]. Furthermore, expression of synaptophysin, an important structural protein in the synaptic vesicle membrane [65], increased after NP treatment in the synaptosome fractions as well as in cultured neurons. This increase is likely due to the accumulation of synaptic vesicles in the synaptic terminals. These data are similar to those observed for silver nanoparticles, which after being deposited in neuronal cell bodies, induced ultrastructural changes in the synapse and altered synapsin I and synaptophysin protein levels in the hippocampus and cortex of rats [66]. These findings suggest that presynaptic terminals are susceptible to NPs.

Collectively, this study implicates SiO_2_-NPs in the development of neurodegeneration-like changes in behavior, neuropathology, and synapse. However, it is also important to consider the mechanism underlying these changes. In previous studies, SiO_2_-NPs have been shown to activate MAPKs in multiple cell types and elicit a variety of responses. For example, SiO_2_-NPs treatment induced proliferation in human adipose tissue-derived stem cells via activation of ERK [67]. In bronchial epithelial cells [68] and macrophages [69], SiO_2_-NPs also activated ERK, leading to inflammation and oxidative stress. In neurodegenerative disease, chronic activation of ERK has been found to mediate neurotoxicity [70], neuroinflammation [71], and tau hyperphosphorylation [4]. In the current study, we also observed prolonged activation of ERK and JNK accompanied by neuropathology and neuroinflammation following NP exposure, suggesting that activation of these MAPKs may play a key role in the adverse effects of NP in the brain. Furthermore, ERK activation also appears to regulate presynaptic terminal function and inhibits exocytosis in primary cortical neuron cultures [72]. To study these effects in our NP-exposed primary neuronal cultures, we pre-treated the neurons with U0126 or SP600125. U0126 is a potent and selective inhibitor of MEK1 and MEK2 kinases [73] and is one of the most widely used inhibitors of the Ras/Raf/MEK/ERK signaling pathway [74], while SP600125 is a reversible, ATP-competitive inhibitor of JNK1/2 [75]. Our data show that pharmacological inhibition of ERK activation prevented the NP-mediated decrease in exocytosis in the cultured neurons. These results strongly agree with previous findings and highlight a distinct regulatory role for ERK in the synapse. Taken together, it appears that the effects of SiO_2_-NPs on behavior, neuropathology, and synaptic function are, at least in part, regulated by ERK activation.

## Conclusions

Taken together, our study provides compelling evidence demonstrating that long-term NP exposure affects cognitive functions and mood, induces neurodegeneration-like changes in the brain, and alters synapse. These changes also appear to be related to the regulation of MAPKs activation. To our knowledge, this longitudinal study is the first published indication that 2 months of intranasal exposure to NP affects multiple aspects in the brains of young adult mice, ultimately leading to neurodegeneration. Therefore, SiO_2_-NPs, which are both environmental-enriched and broadly applied, may be a risk factor for neurodegenerative diseases even in young adult individuals with a higher resistance to foreign stimulus.

## Methods

### Synthesis and characterization of NPs

In the present study, FITC-SiO_2_-NPs were used to represent amorphous SiO_2_-NPs. The FITC-SiO_2_-NPs were synthesized with the Stöber method [76, 77]. Using this method, the FITC was linked to the SiO_2_-NPs via an interaction between the modified amine group of the FITC and the silane group on the SiO_2_-NPs, thereby trapping the FITC in the silica layer [78]. The full synthesis procedure utilized for the construction of the FITC-SiO_2_-NPs can be found in the Additional file 1. All of the chemicals used in this study were purchased from Sigma-Aldrich (St. Louis, Missouri, USA), unless otherwise noted.

The morphology of the NP was characterized with TEM (Tecnai G2 20 S-TWIN TEM, FEI, Oregon, US). The TEM images were then used to determine the average diameter of the FITC-SiO_2_-NPs using Image J software (http://rsb.info.nih.gov/ij/). The hydrodynamic diameter and zeta potential of the NP in PBS (the vehicle used to administer the NP to the mice), neuronal culture medium (1.6% fetal bovine serum), and water were measured by DLS using a low volume disposable sizing cuvette (ZEN0112) on a Zetasizer Nano (Malvern, Worcestershire, UK). Before each measurement, the NP were sonicated with a Fisher Sonic Dismembrator (Model 300, Markham, Ontario, CA) at 35% amplification for 15 min. The sonicated samples were then diluted to 1 mg/ml with distilled water and filtered through a 0.22 μm nylon membrane filter. The z-averaged hydrodynamic mean diameter, zeta potential, and polydispersity of the samples were calculated using software provided by Malvern (Worcestershire, UK).

### Animals, treatment, and tissue sampling

Male C57BL/6 N mice (3 months old) were group-housed in cages with regular rodent chow and tap water provided ad libitum in the clean area of the Laboratory Animal Unit at The University of Hong Kong. The light cycle of the animals was from 7 am to 7 pm, and all of the experimental handling, such as administration and behavioral tests, took place during this light cycle. To minimize NP aggregation in the aqueous medium, the NP were sonicated (Fisher Sonic Dismembrator, Model 300) at 35% amplification for 15 min right before intranasal administration. To minimize the disturbance from handling during administration to the results of behavioral tests, the administration was undertaken after behavioral test by the same operator at the same time of the day in the same room. The protocols used in this study were approved by the Committee on the use of Live Animals in Teaching and Research, The University of Hong Kong. In total, 40 animals were randomly assigned to two groups: PBS-treated (control) and NP-treated.

The recommended maximum level of amorphous silica in the workplace varies from 2 mg/m^3^ in New Jersey [79] to the 10 mg/m^3^ limit set by the American Conference of Governmental Industrial Hygienists, an organization which sets workplace chemical exposure limitations [80]. Therefore, depending on the job location, a 60 kg worker present in the workplace for 8 h without proper protection will breathe in approximately 6.7 m^3^ of air containing 13.3 to 66.7 mg of amorphous silica, resulting in an approximate dose of 0.22 to 1.1 mg/kg/day. To reflect these levels, the dosage should be 2.7 to 13.53 mg/kg in mice based on body surface area [81]. In the present study, the dosage for mice was set at the median value, 8 mg/kg.

Thus, mice in the control and NP groups were administered 20 μl of vehicle (PBS) or FITC-SiO_2_-NPs (8 mg/kg, 10 mg/ml in PBS), respectively, through bilateral intranasal instillation. The intranasal instillation was done with a 20 μl pipette with an extended-end tip. The fully conscious mouse was handled with its head up, and the end of the pipette tip was placed near the nostril. Each instillation was separated into four smaller aliquots to ensure minimal leakage to the mouth. The treatment duration was 81 days.

After perfusion with normal saline, the brain was dissected and the frontal cortex and hippocampus were isolated [82]. For synaptosome extraction, the tissue samples were extracted with Syn-PER reagent (Thermo Fisher, Waltham, Massachusetts, USA) supplemented with protease and phosphatase inhibitors according to the manufacturer’s protocol. For total lysate extraction, the tissue samples were homogenized with RIPA buffer (Cell Signaling Technology, Danvers, Massachusetts, USA) supplemented with protease and phosphatase inhibitors. RNA was extracted from the tissue samples with RNAiso Plus reagent (Takara, Kusatsu, Shiga, Japan) and cDNA was synthesized using PrimeScript RT Master Mix (Takara, Kusatsu, Shiga, Japan) as previously described [83]. To prepare frozen embedded tissue, half of the perfused brain was post-fixed in 4% paraformaldehyde (PFA) for 72 h and then soaked in 30% sucrose in phosphate buffer (PB) until saturated. The tissues were then embedded in tissue freezing medium (Leica, Wetzlar, Germany) and sectioned with a cryostat (Leica, Wetzlar, Germany). The sections were stored in PBS at 4 °C until free-floating staining was performed.

### Behavioral tests

#### Grouping and behavioral test arrangement

During treatment (at 1 month and 2 months), mice were tested with a battery of behavioral tests, as shown in Fig. 2a. The order of these behavioral tests was from the least to the most stressful to the animals. Notably, some behavioral tests can be repeated, such as the novel object recognition test when different pairs of objects are used, while others, such as the Morris water maze test, cannot be repeated because the outcome may be affected by the previous experience. For this reason, the control and NP-treated mice tested at 1 month (*n* = 8 per group) were not tested again for some experiments, while a second set of control and NP-treated mice (*n* = 12 per group) were tested only at the 2 month time point.

To study cognition in the treated mice, we used the novel object recognition test with a 24 h inter-session interval to study their memory and the Morris water maze test to study their spatial learning and memory. Furthermore, since the NP were administered via intranasal instillation, it is possible that cardiopulmonary function could be affected, making it essential to evaluate any aberrant effects on motor function. We used the open field test and the accelerating rotarod test to study the locomotor and motor functions after NPs exposure, respectively. Moreover, it was also important to understand any psychological changes associated with treatment. Social interaction activity is an important aspect affected in  anxiety and depression, both of which are commonly observed during neurodegeneration [23]. To elucidate any anxiety-related symptoms, we use the elevated plus maze test, the golden standard for measuring anxiety [43]. In addition, central area duration during the open field test can also serve as an indicator for anxiety [84]. The forced swimming test and tail suspension test, also known as ‘despair tests’, were used to test for depression, which is measured in relation to the immobility time during the tests.

The protocols utilized for the open field test [84], elevated plus maze test [43], novel object recognition test [44], social interaction test [83], accelerating rotarod test [83], Morris water maze test [85], forced swimming test [86], and tail suspension test [87] were similar to those reported in the literature. A detailed protocol for each is provided in the Additional file 1.

### Primary culture of cortical neurons

Primary cultures of cortical neurons were prepared from embryonic day 18 (E18) Sprague-Dawley rats similar to a previous report [88]. A detailed protocol is provided in the Additional file 1. This protocol was approved by the Committee on the use of Live Animals in Teaching and Research at The University of Hong Kong.

After 12 days in culture, the cortical neurons were exposed to 2 ml of medium (2:1 ratio of complete Neurobasal medium and Minimum Essential medium supplemented with 1.6% fetal bovine serum) with PBS or NP at a concentration of 10 μg/ml. Notably, the NP were sonicated immediately prior to use to avoid aggregation, as described above. The cells were incubated in a 37 °C inbubator with 5% CO_2_ for 48 h and then analyzed using the FM dye assay or immunocytochemical staining. To further study the role of ERK and JNK activation in the NP-mediated changes, neurons were pre-treated with 1 μM of either U0126 or JNKi 1 h before exposure to NP for 48 h. These treatments were performed according to the manufacturer’s instructions for U0126 as well as previous reports for JNKi [89]. After treatment with NPs, the neuronal cultures were analyzed using the FM dye assay.

### FM dye assay

FM dyes label the lipid membrane and have, therefore, been used to monitor endocytosis and exocytosis in synaptic vesicles in both cell culture and synaptosomal fractions of brain tissues. With this assay, the gain and loss of fluorescence demonstrates the efficiency of endocytosis and exocytosis in the synapse, respectively.

The freshly prepared synaptosome fractions were quantified and diluted in Hanks’ balanced salt solution (HBSS, Gibco, US), and then incubated with 100 μM of FM1–43 dye (Thermo Fisher, Waltham, Massachusetts, USA) and 30 mM KCl at room temperature for 15 min to activate endocytosis and to induce dye uptake. Then, the mixture was centrifuged at 15000×g for 5 min at 4 °C and washed with HBSS twice. Each pellet was resuspended in 350 μl of HBSS, and 100 μl of the suspension was added to a 96-well plate (in triplicate) for analysis on a plate-reader (Perkin Elmer, Waltham, Massachusetts, USA) at an excitation/emission of 530/600 nm. This was designated the endocytosis reading (F_endo_). Upon the finishing this analysis, the synaptosome suspensions in the wells were collected and incubated with 30 mM KCl for 15 min at room temperature to activate exocytosis, followed by centrifugation, washing, and resuspension as described above. The fluorescence analysis was designated the exocytosis reading (F_exo_). Exocytosis function of the sample of synapses was calculated as (F_endo_ – F_exo_)/F_endo_. The results of this analysis for both the endocytosis and exocytosis readings are shown as the fold change compared to the control.

The FM dye assay protocol used to analyze the primary neuronal cultures was similar to that previous reported [90]. Briefly, FM4-64FX (LifeTechnologies, Carlsbad, California, USA) fluorescent dye was dissolved in HBSS to achieve a working concentration of 5 μM. The primary cortical neurons were cultured in 6-well plates on coverslips, two per well. Each well represented one treatment condition. After treatment, the culture medium was removed and the neurons were briefly washed with HBSS, followed by stimulation with 100 mM KCl for 5 min to induce endocytosis. After washing with HBSS, the cells were incubated with dye for 30 min at 37 °C. Then, the cells were washed and one group was fixed with 4% PFA for 13 min. The remaining neurons were then stimulated with 100 mM potassium chloride for 10 min to induce exocytosis and then fixed with 4% PFA after washing. The coverslips were mounted on microscope slides using ProLong® Gold antifade mounting medium (LifeTechnologies, Carlsbad, California, USA). Images were taken with a LSM510-meta laser scanning confocal microscope (Carl Zeiss, Oberkochen, Germany) and quantified using ImageJ (http://rsb.info.nih.gov/ij/). The average fluorescence intensity in both the endocytosis (F_endo_) and exocytosis (F_exo_) steps were normalized to the control group to show the change in function after NP exposure.

### Western blot

Western-blot analysis was performed as previously reported [91]. The full protocol and antibodies using in this study are listed in the Additional file 1.

### Real time-PCR (RT-PCR)

RT-PCR was performed using SYBR Premix Ex Taq™ II kit (Takara, Shiga, Japan) and employing the StepOnePlus Real-Time PCR System (Applied Biosystems, Thermo Fisher Scientific, USA) as previously reported [83]. The primers for IL-1β, IL-6 and GAPDH were the same as in literature [83].

### Immunofluorescence, immunohistochemistry, and image analysis

In the primary cortical neuron cell cultures, the expression of various synaptic proteins was studied using immunocytochemical staining and imaged with an LSM510-meta laser scanning confocal microscope (Carl Zeiss, Oberkochen, Germany) equipped with a 63X oil objective lens. Five representative neurons were imaged on each coverslip. The fluorescence intensity of the images was quantified with Image J. Alternatively, the brain tissue sections from every 9 sections of the mPFC (bregma + 2.68 mm to + 1.54 mm) and every 15 sections of the hippocampus (bregma − 1.34 mm to − 2.46 mm) were evaluated using immunohistochemistry or immunofluorescence. A total of three non-overlapping fields in the mPFC as well as the CA1, CA3, and DG in the hippocampus were imaged. The detailed protocols and antibodies used in these analyses are shown in the Additional file 1.

Notably, the deposition of NP was studied using immunofluorescent staining of free-floating frozen brain sections from three representative animals per group, followed by confocal microscopy (LSM 700, Carl Zeiss, Oberkochen, Germany). Microglia in the mPFC and hippocampus were labeled with an Iba-1 antibody and imaged by confocal microscopy. Quantification of microglia cells in the area of interest was done with Image J. The detailed protocol for measuring the number and area of the stained cells can be found in the Additional file 1. Nissl staining was also performed to reveal granules, known as Nissl bodies, in the neuronal cytoplasm using a previously reported protocol [92]. A Zeiss Axiophot microscope (Carl Zeiss, Thornwood, US) with a 20X objective lens and a SPOT RT3TM camera (SPOT Imaging Solutions, Sterling Heights, US) was used to acquire the images. Scale bar for confocal microscopic images and light field images was added by ZEN (https://www.zeiss.com.cn/microscopy/downloads/zen.html#inpagetabs-0) and AxioVision (https://www.zeiss.com/microscopy/int/downloads/axiovision-downloads.html), respectively. Notably, the researcher processing all the images was blinded to the treatment groups to avoid bias.

### Statistical analysis

All data, including that from the behavioral tests, western blot analysis, immunohistochemistry, and in vitro studies, were first examined by the Shapiro-Wilk normality test to test for normal distribution. If the data was normally distributed, a two-tailed Student’s *t*-test or one-way ANOVA was used for statistical analysis. If the data were not normally distributed, the Mann-Whitney test was used. To analyze the data obtained for the Morris water maze test training phase or the rotarod test, a repeated-measured ANOVA was conducted with treatment as the within-subject factor and time as the between-subject factor. All statistical analyses were conducted with Prism 6 software (GraphPad Software, La Jolla, California, USA). All results are presented as the means ± standard error of the mean (s.e.m.). In all cases, a *p*-value less than 0.05 was considered statistically significant.

## Additional file

Additional file 1: (**Methods.** Effects of FITC-SiO_2_-NPs on behavior in mice (**Figure S1.**) Effects of FITC-SiO_2_-NPs on phosphorylations of p38 and CREB and its effectors in total lysates of frontal cortex and hippocampus (**Figure S2.**) Protein levels of synaptophysin and synapsin I in the primary culture of cortical neurons exposed to NP for 48h as analyzed by Western blot (**Figure S3.**) DOCX 1064 kb)

## Abbreviations

AD: Alzheimer’s disease
CA: Cornu ammonis area
COX-2: Cyclooxygenase 2
CREB: cAMP responsive element binding protein
DG: Dentate gyrus
DLS: Dynamic light scattering
ERK: Extracellular signal-regulated kinases
FITC: Fluorescein isothiocyanate
FTD: Frontotemporal dementia
HBSS: Hank’s balanced salt solution
IL: Interleukin
JNK: c-Jun N-terminal kinases
MAPKs: Mitogen-activated protein kinases
mPFC: Medial prefrontal cortex
PBS: Phosphate buffered saline
PD: Parkinson’s disease
PM: Particulate matter
RT-PCR: Real time-PCR
SiO_2_-NPs: Silica nanoparticles
TEM: Transmission electron microscopy
