# Supplementary material for: Silica nanoparticles induce neurodegeneration-like changes in behavior, neuropathology, and affect synapse through MAPK activation
Source: Part Fibre Toxicol. 2018 Jul 3;15:28. doi: 10.1186/s12989-018-0263-3 (PMC6029039; doi:10.1186/s12989-018-0263-3)
Supplement: Supplementary file 1 — (Methods. Effects of FITC-SiO2-NPs on behavior in mice (Figure S1.) Effects of FITC-SiO2-NPs on phosphorylations of p38 and CREB and its effectors in total lysates of frontal cortex and hippocampus (Figure S2.) Protein levels of synaptophysin and synapsin I in the primary culture of cortical neurons exposed to NP for 48h as analyzed by Western blot (Figure S3.) DOCX 1064 kb) [file 12989_2018_263_MOESM1_ESM.docx]

**Supplementary material**

**Methods**

**Preparation of FITC-SiO_2_-NPs**

All the chemicals were purchased from Sigma-Aldrich (St. Louis, Missouri, USA). Briefly, FITC and ethanolic 3-aminopropyltriethoxysilane (APTES) solution (the amount of APTES was 9 mol % of TEOS and was excess than that of FITC) was stirred in the dark overnight to form N-1-(3-tri-ethoxysilylpropyl)-N’-fluoresceyl thiourea (FITC-APTES). Separately, Silica particles were prepared by adding a premixed ethanol solution (25 ml) containing ammonium hydroxide (1.5 ml) and TEOS solution (2 ml) under stirring for 1 h. Then the FITC-APTES was added and the solution was aged at room temperature for 24 h. The product was washed with ethanol and sterile phosphate buffered saline (PBS), and was eventually stored in sterile PBS. To measure the concentration of FITC-SiO_2_-NPs, 1 ml of FITC-SiO_2_-NPs suspension was dried in 60°C oven overnight and weighed.

**Primary culture of cortical neurons**

The cortices were isolated from the brains of E18 fetus and were then dissociated in 1x PBS solution supplemented with 18 mM glucose and seeded on 35 mm culture dish (Iwaki) with cover slips (Thermo Scientific) which are coated with poly-L-lysine (25 µg/ml, Sigma) solution in water overnight. Cells were seeded at 0.75 x 10^5^ cells per cover slip for FM dye and immunocytochemistry. Cells were cultured in a 2:1 mixed medium composed of Neurobasal (NB) medium (Gibco) supplemented with B-27, 2 mM L-glutamine, 10 μg/ml penicillin/streptomycin and 25 μM β-mercaptoethanol and supplemented MEM medium. Deoxyfluoridine was added to inhibit the growth of non-neuronal cells 1 day after seeding. Five hundred μl of medium were swabbed by 500 μl fresh complete NB medium at day 4 and 8 after seeding. Neurons were kept in a humidified incubator at 37°C and 5% CO_2_ for 12 days prior to the 48h-treatment.

**Behavior tests**

All the behavioral experiments took place in a behavioral room without other stimulation including direct bright light to the experimental apparatus, odor, noise and other irrelevant animals and equipped with video recording system that is out of the sight of the animals. Before the start of experiment, all the animals were habituated in their home cages to the experimental environment in the behavioral room for 30 min. The experimental apparatus that may have olfactory trace left by other mice, which includes open field arena, elevated plus maze, social interaction chambers, objects in the novel object recognition test, and rotarod, was cleaned by tap water and wiped with paper towel sprayed with 70% ethanol to remove the odor before and after the experiment. The water in the cylinder used in forced swimming test was replaced for each subject mouse. The mice were returned to home cage after finishing video recording. All mice were returned to animal unit where they were kept in home cages once the experiment is finished. The order of the animals in the tests was counterbalanced between treatment groups and the treatment groups were blinded by the experimenter.

***Open field test***

The open field test was conducted in 60 cm (L) x 60 cm (W) x 40 cm (H) nontransparent white plastic arena, which is placed in a dim-lighted behavior room. At the beginning of the experiment, the subject animal was gently put in the arena, facing to the middle of a wall of the arena. Afterwards, the experimenter left the behavioral room to avoid disturbance. The behavior of the mouse inside the open field arena was recorded for 10 min. For data analysis, the open field arena was divided by a 4 x 4 imaginary grid, and the number of lines crossed by the mouse in the arena was counted from the video as a measurement to the locomotor function; central area duration was also timed as an indicator of anxiety.

***Elevated plus maze***

The apparatus used for the elevated plus maze test was in the configuration of a + and comprises two open arms (30 x 5 x 0.5 cm) across from each other and perpendicular to two closed arms (30 x 5 x 16 cm) with a center platform (5 x 5 x 0.5 cm). Each arm of the maze is attached to sturdy metal legs such that it is elevated 40 cm off of the ground. At the beginning of the test, the subject mouse was taken out of its home cage and placed at the junction of the open and closed arms, facing to the open arm that is opposite to the experimenter. The video was recorded for 5 min. The times of entries and duration in open arms and closed arms were measured according to video.

***Novel object recognition test***

The protocol applied is composed of 4 days, which includes 2 days of habituation, 1 day of familiarization and 1 day of testing. Habituation means 10 min in open field arena as previously described. Familiarization means 10 min in the open field arena with two identical objects placed 5 cm away from the side and 7 cm away from the top of the arena. Test means 10 min in the open field arena with one object replaced by a novel object, but the other object and the position of the object remains the same. The novel object and the position of the object were counterbalanced to avoid biases. The experiment took place in the same time during the 4-day experiment to assure the test is 24h later than familiarization. During each day, the subject mouse was introduced into the arena in the same way as open field test, and the behavior of the mouse will be recorded for 10 min. After 10 min, the mouse will be returned back to the home cage for rest. The amount of time the mouse spent interacting with both objects was determined from the videotape and the ratio of time interacting with the novel object to the time interacting with the old object was calculated. An interaction between the animal and the object is defined by if its nose is pointing towards the object within 2 cm.

***Social interaction test***

Briefly, two identical transparent chambers (8 cm (L) x 6 cm (W) x 12 (H)) with holes on the surface were placed by the middle of the side in the open field arena. The subject mouse was put into the arena for habituation for 3 min. Then the subject mouse was return to the home cage. The arena was briefly cleaned with paper towel and a novel juvenile (4 weeks old) helper at the same gender (male) as the subject animal was placed in one of the chamber and placed back to the arena. The subject mouse was introduced back to the arena and video was recorded for 3 min. After the recording is finished, both the subject animal and helper were returned back to their home cages. The amount of time the subject mouse spent interacting with both chambers was counted from the videotape and the ratio of time interacting with the chamber with helper to the time interacting with the empty chamber was calculated. An interaction between the animal and the chamber is defined by if its nose is pointing towards the object within 2 cm.

***Accelerating rotarod test***

The rotarod test was used to test the motor function at three time points of the treatment, i.e before the treatment, 1 month treatment and 2 month treatment. The training of rotarod was done before the treatment, which is three-day training. On each day, the animals had three trials. During each trial, the mice were placed on the rotarod, facing to the wall. The rotarod was programmed to accelerate as following: starting from 4 rpm and accelerating to 40 rpm within 5 min. The time that the animal stayed on the rod was recorded, and the average time of the three trials on the third day was used as the baseline of the motor function. On the 1 month and 2 month treatment time points, the rotarod test was done for 1 day, which has 3 trials with the same accelerating programming. The time that the animal stayed on the rod was recorded and the average time was used.

***Morris water maze test***

The water maze was a circular pool (120 cm in diameter, 60 cm deep) divided into 4 equal imaginary quadrants for data analysis. The water temperature was maintained between 21 and 23^◦^C and the water was colored into white by milk powder. A white circular platform 10 cm in diameter was placed 1 cm beneath the surface of the water. The platform was hidden from the view of animal during acquisition phase. The swimming patterns of the mice was recorded with a video camera mounted above the center of the pool. The water maze was equipped with visual cues hanging on the walls to provide spatial cues. The acquisition phase was carried out for 5 consecutive days. On each training day/session, each mouse received 4 consecutive training trials with 4 different starting locations. The mouse was left in water for 60s or until it found the hidden platform and stayed on it for 10s. For those who could not find the platform within 60 s would be guided to the platform by the experimenter and being left on it for 10s. At the end of each trial, the mouse was gently dried with paper towel and put into home cage for rest. The cage was warmed with infar-red lamp to guarantee the warmth of the animals. The time latency to reach the platform was recorded.

Day 6 of MWM was called probe phase, which was to assess spatial retention. During probe test, the platform was removed, and the animal was placed into the water from the point that was the opposite of where the platform used to be. The animal was left in water for 60 s and then taken out, dried and returned back to home cage. The percentage of time spent in the target quadrant (where the platform was located during hidden platform training), and the times crossing the original platform was measured.

***Forced swimming test***

The cylindrical tanks (30 cm height, 20 cm diameters) used in forced swimming test was filled with 15 cm deep of tap water set at the room temperature (23-25°C) and the water level was marked on the tank walls. Start video recording before placing the animals into the water tanks. Holding by its tail, the animal was gently and slowly placed in the water. Once the mouse was in the water, slowly release the tail and start the countdown for six minutes. After time was up, the mouse was gently dried and put into home cage with a heater nearby.

***Tail suspension test***

The animal was hanging with a tape on its tail where 1 cm away from the tip. The approximate distance between the mouse's nose and the apparatus floor was 20-25 cm. Record the video for 6 min and then released the animal back to home cage when time was up.

**Western blot**

Briefly, equal amount of protein was applied for electrophoresis in SDS-PAGE gel and then transferred to polyvinylidene fluoride membranes (Bio-Rad, Hercules, California, USA). The membranes were than blocked with 5% nonfat milk (Bio-Rad, Hercules, California, USA) in TBST (0.1% tween 20 in TBS) for 1 h and then incubated with primary antibodies listed below. After washing, the membranes were incubated with horseradish peroxidase conjugated secondary antibody for 2 h in room temperature. Immunoreactivity was detected using WesternBright ECL (Advansta, Menlo Park, California, USA) in MyECL imager (Thermo Fisher, Waltham, Massachusetts, USA). Since some markers which have similar molecular weight but their primary antibodies have different hosts, such as p-tau S396 and α-tubulin, the membrane was first developed with the one with weaker signal and then stripped to re-probe another one.

The primary antibodies used in the supplementary materials and the conditions are as following:

| Synaptophysin | Millipore | 1:20000 | r.t., 2h |
| --- | --- | --- | --- |
| Synapsin I | Thermo Fisher | 1:30000 | r.t., 2h |
| p-tau S396 | Millipore | 1:3000 | r.t., 2h |
| COX-2 | Santa Cruz | 1:1000 | 4 °C over night |
| α-tubulin | Sigma-Aldrich | 1:30000 | r.t., 2h |
| p-ERK | CST | 1:1000 | 4 °C over night |
| ERK | CST | 1:1000 | 4 °C over night |
| p-JNK | CST | 1:1000 | 4 °C over night |
| JNK | CST | 1:1000 | 4 °C over night |
| p-p38 | CST | 1:1000 | 4 °C over night |
| p38 | CST | 1:1000 | 4 °C over night |
| p-CREB | CST | 1:1000 | 4 °C over night |
| CREB | Millipore | 1:1000 | 4 °C over night |
| c-fos | Calbiochem | 1:1000 | 4 °C over night |
| BDNF | Santa Cruz Biotech. | 1:1000 | 4 °C over night |
| GAPDH | Sigma-Aldrich | 1:3000 | r.t., 2h |

**Immunocytochemistry & immunohistochemistry**

***Immunocytochemistry***

For immunocytochemical staining, primary culture of cortical neurons cultured on coverslips were washed by TBS for 5 min, and then fixed with 4% PFA for 15 min, permeabilized with 0.1% Triton X-100 in TBS for 7 min, and blocked with 5% bovine serum albumin in TBS for 1 h. Incubation of primary antibody was done for 1 h at room temperature at 1:400 dilution for the following antibodies: synaptophysin (Millipore, Billerica, Massachusetts, USA) and synapsin I (Thermo Fisher, Waltham, Massachusetts, USA). Then, the cells were incubated with secondary antibody (anti-rabbit or mouse, Alexa-fluor 568, 1:400, Molecular Probes, Eugene, Oregon, USA) and mounted on microscope slides (Thermo Fisher, Waltham, Massachusetts, USA) using ProLong^®^ Gold antifade mounting medium (LifeTechnologies, Carlsbad, California, USA) and imaged using the LSM510-meta laser scanning confocal microscope (Carl Zeiss, Oberkochen, Germany). Images were analyzed using ImageJ. The average fluorescent intensity was normalized with control group.

***Immunofluorescence***

The fluorescent immunohistochemistry was done using frozen brain samples in a free-floating way. The picked sections were blocked with 10% normal goat serum in PBS for 2 h, and then incubated with FITC primary antibody (1:100, Thermo Fisher, Waltham, Massachusetts, USA) and then primary antibody of NeuN (1:200, Millipore, Billerica, Massachusetts, USA) both overnight in 4°C, or only with Iba-1 antibody (1:100, Wako, Japan), and then incubated with (anti-rabbit or mouse, Alexa-fluor 488 or 568, 1:400, Thermo Fisher, Waltham, Massachusetts, USA) for 2 h in room temperature, followed by incubating with 3 mM DAPI (Thermo Fisher, Waltham, Massachusetts, USA) for 20 min in room temperature. After washed with PBS, the sections were mounted on Superfrost^TM^ plus microscope slides (Thermo Fisher, Waltham, Massachusetts, USA) until dry and then mounted with coverslip using mounting medium (DAKO, Santa Clara, California, USA). After the mounting medium was dried, the sections were imaged using LSM700 confocal microscopy (Carl Zeiss, Oberkochen, Germany).

***Nissl staining***

Briefly, the coronal brain sections from both of the treatment groups were mounted on the same slide and dried. Then they were stained with 0.1% (w/v) cresyl violet acetate for 5 min and rinsed with water to remove the extra dye. After differentiated in 95% ethanol, the slides were then dehydrated through graded concentrations of ethanol, and cleared in xylene before mounting.

***Image acquire and analysis***

To prevent disturbance from the microscope such as the laser, all the groups in the same experiment were imaged in parallel. Confocal microscopy was used to acquire fluorescent images, and settings were aimed to acquire the strongest signal while block the autofluorescence and minimize noise. To minimize the interference of the unrelated regions in the brain sections or other neurons in the primary neuronal culture, area of interest was selected during imaging.

The images from Iba-1 staining were analyzed by Image J similar to literature. Firstly, the area of interest was selected, such as the Nissl staining images in CA1, CA3 or DG in the hippocampus. Since the images of Iba-1 staining were in higher magnification, the area of interest was selected during imaging. Then, the threshold of the image was determined to highlight the positive staining, such as Nissl bodies presented as the darker stained dots in neurons, and cell body of microglial cells labeled with Iba-1. Then the ImageJ gave the number and area of the selected dots by clicking ‘Analyze’ and then ‘Analyze particles’. The experimenter performing the image process was blinded to the treatment group to avoid bias. Nissl bodies number/area and microglia cell number/graph was exported to Excel for further analysis.

Fluorescent intensity of primary neurons in the FM dye assay and in the immunofluorescence for synaptic proteins were measured by image J in the area of interest. After subtracting background, the fluorescent intensity of the images of an individual neuron with similar number of neurites was measured. All the images in the same experiment had the same experimental setting. The fluorescent intensity of each group were normalized to that of control.

**Results**

**Fig. S1** Effects of FITC-SiO_2_-NPs on behavior in mice. (**a**) Open field test were used to study the locomotor function in mice. Unpaired Student’s t-test and unpaired Mann-Whitney test was used to analyze data for 1-month and 2-month, respectively. (**b**) Rotarod test were used to study whether the motor function of mice changed through out the treatment. Data was analyzed by repeated-measured two-way ANOVA. (**c**) The central area duration in open field can be used as an indicator of anxiety. Unpaired Mann-Whitney test was used to analyze data in each time point. Forced swimming test (**d**) and tail suspension test (**e**) were used to study the depression in mice. Data in these two tests was analyzed by unpaired Student’s t-test. (**f** & **g**) Preference of target quardrate in probe test of Morris water maze test indicated the searching strategy the mice applied. Unpaired Student’s t-test was used to analyze data in each time point.

CREB activation can activate the expressions of c-fos, an immediate early gene which is used for mapping neuronal activity, and the brain-derived neurotrophic factor (BDNF), a pro-survival factor. However, we did not find any change in the phosphorylation of CREB, or in the expressions of c-fos and BDNF in the frontal cortex (Fig. S2), indicating that CREB signaling was not activated. Although significant increase in phosphorylation of CREB was found in the hippocampus of NPs-exposed mice, the protein levels of c-fos and BDNF were not changed accordingly, indicating the change in the CREB phosphorylation in the hippocampus might lead to no effect on the behavior.


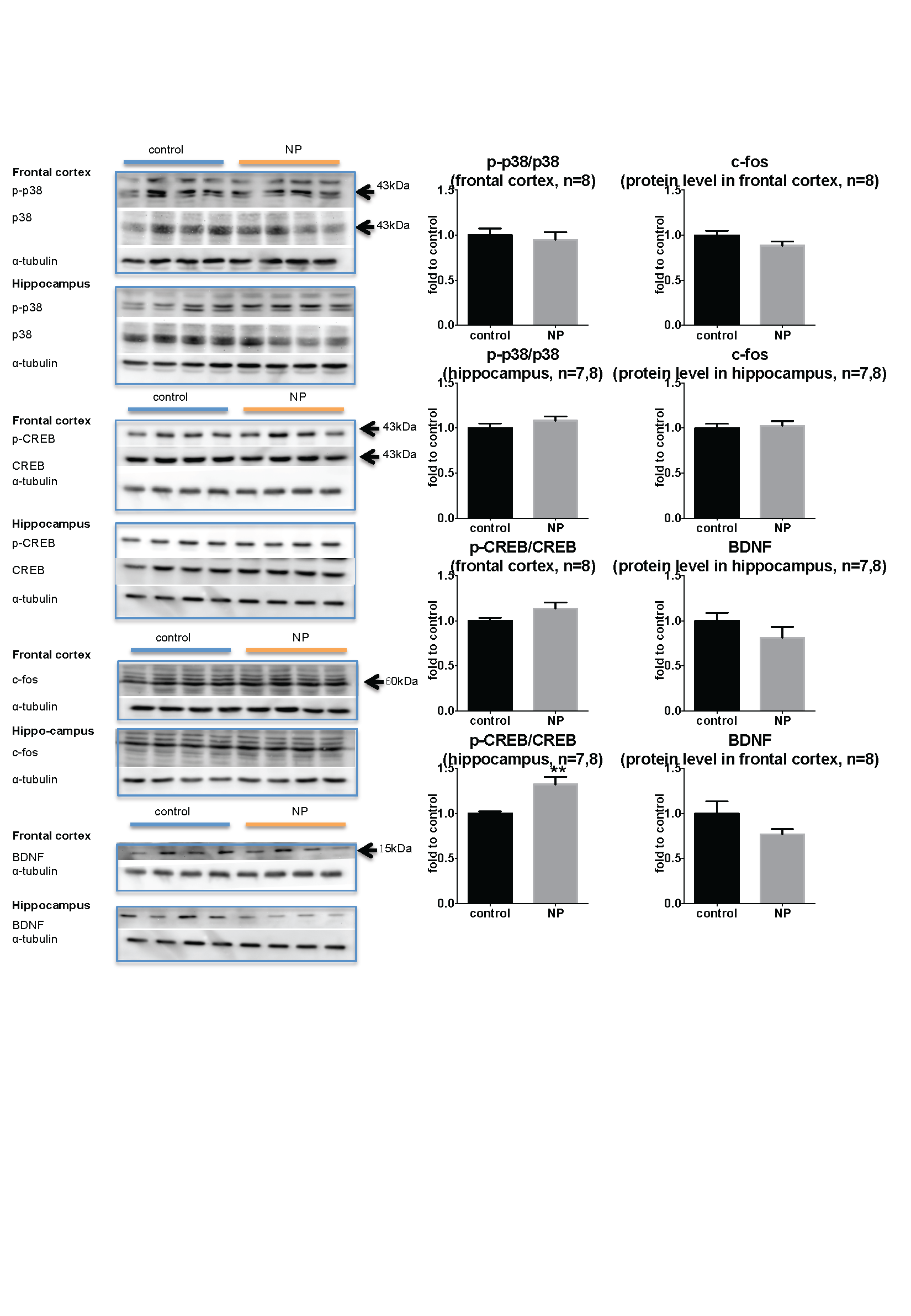


**Fig. S2** Effects of FITC-SiO_2_-NPs on phosphorylations of p38 and CREB and its effectors in total lysates of frontal cortex and hippocampus. Data were analyzed by unpaired Student’s *t*-test for each marker at each region. n=8 for all groups, except n=7 for hippocampus from control group. 4 representative bands were shown.


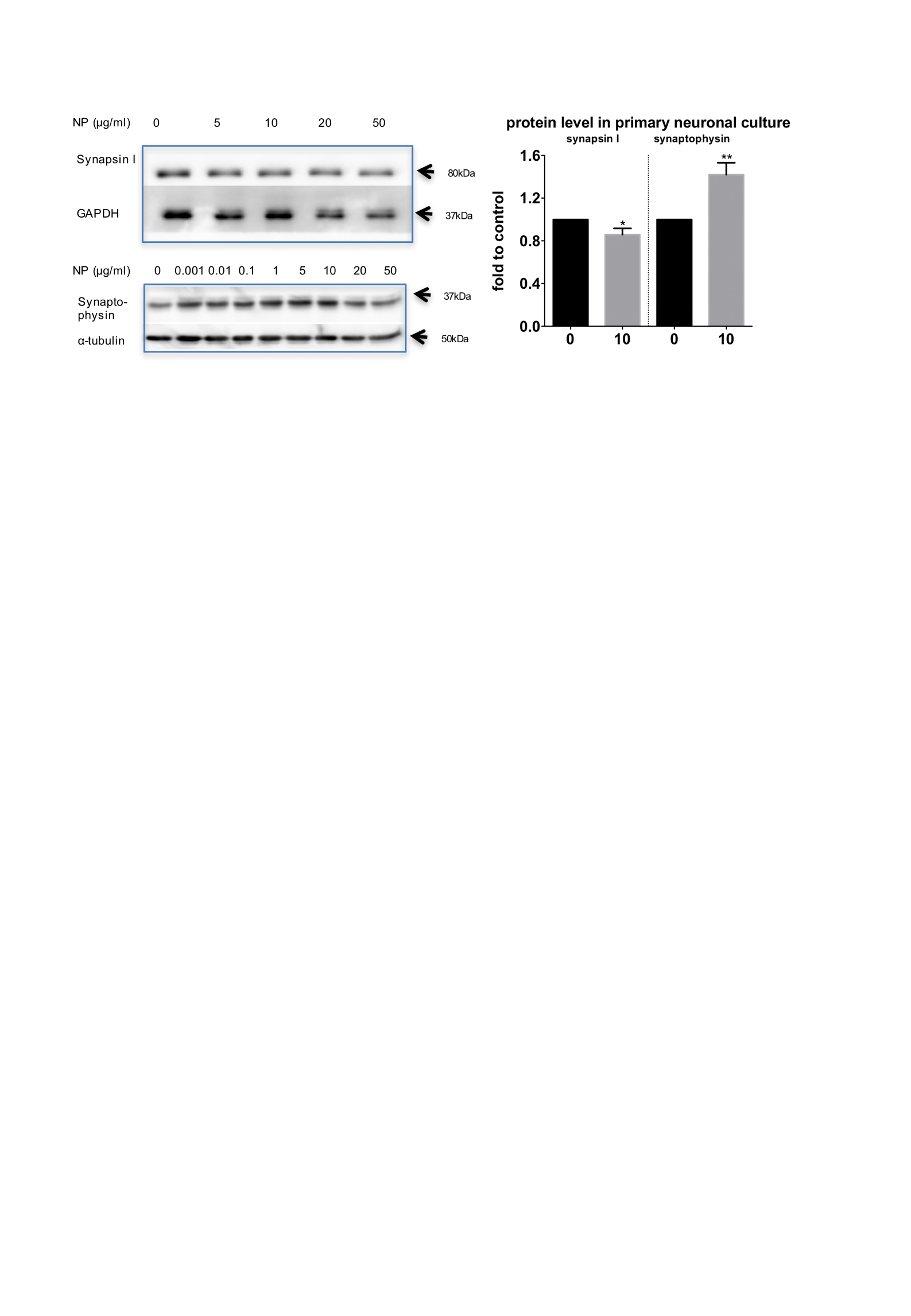


**Fig. S3** Protein levels of synaptophysin and synapsin I in the primary culture of cortical neurons exposed to NP for 48h as analyzed by Western blot. Primary culture of cortical neurons was exposed to vehicle (PBS) or NP at different dosages for 48h before total lysate was extracted. Same as the results in the immunofluorescence, NP at dosage of 10 μg/ml significantly decreased the protein level of synapsin I and increased the protein level of synaptophysin in the total lysate of primary culture of cortical neurons. n = 6 or 4 independent batch of samples for synapsin I and synaptophysin, respectively. Unpaired Student’s *t*-test was used as statistical analysis. * , ** means p < 0.05 or 0.01 compared to control, respectively.
